# Address of the Retiring President of the Buffalo Dental Association

**Published:** 1865-07

**Authors:** E. Hayes


### ADDRESS OF THE RETIRING PRESIDENT OF THE BUFFALO DENTAL ASSOCIATION,

DR. GEO. E. HAYES,

Delivered June 6th, 1865.

*Gentlemen of the Association:*—The time has come, when as your presiding officer I am permitted to give place to a successor, and when by your by-laws you expect something in the form of an address.

I am glad to embrace the opportunity of expressing my thanks for the unvarying kindness and support which has rendered the discharge of the duties of the chair always pleasant and agreeable, and also for the zeal with which you have followed up the objects of the Association. Beyond this, I hardly know what I can say that will be new, useful, or amusing. Since the day our first parents were driven out of Paradise, man has been subject to disease, sickness and death.

The curse which condemned the human race to eat bread in the sweat of the brow, which compels us to labor and toil for our necessary food and clothing, is light, in comparison with the constant, anxious efforts we are compelled to make, to keep off, and to repair the ravages of the destroyer.

In every age of the world, the best talent has been demanded to provide against disease and premature decay, and to render life useful or even tolerable, to that time, when old age welcomes the grave as a final resting place.

With all people, the Doctor has always been an indispensable attendant. His services have commanded alike the wealth of the rich, and the thanks and gratitude of the poor. The cry for help, help, comes up from the whole human race. All alike appeal to him for relief from pain, and for a respite from the dread sentence which assigns all back again to the dust from whence we were taken. It has fallen to our lot, gentlemen, to fight the battle of life in one of the divisions of this great army of Doctors. The position is full of responsibility, and demands our constant and best efforts; but in some respects we may be regarded as a favored class.

While we, like all others, are compelled to earn bread by the sweat of the brow, we are allowed the satisfaction of knowing that we are doing good to our fellows. We may forget or even learn to love the toil in view of the comfort we are enabled to bestow on suffering humanity. As good soldiers, then, is it not our duty to forget self, and look solely to the welfare of those we have in charge, and those who are to follow in our footsteps? If this is so, how then shall we acquit ourselves to the best advantage?

How can we ward off disease from our patients most effectually? How can we prolong their existence in comfort and usefulness to the greatest age, are questions which ought and will press upon the attention of every honest practitioner.

It is well for us, and a beautiful illustration of the goodness of the Creator, that in this our duty and our self-interest are perfectly harmonized. The man who gives his whole thoughts to confer benefits on others, is sure in the end to receive a better reward than he who only studies to fill his own pockets, and is always tormenting himself with the fear that his labor will go unrequited. How, I ask, shall we do this? Can we do it by keeping ourselves close from one another? By husbanding up, as secrets, all the little improvements we may happen to think we make, and allowing their benefits to be doled out to those only, who chance to call on us individually, and at the same time depriving our own patients of all the benefits arising from all the improvements made by our fellows, except such as we can filch and pirate from them, and then perhaps claim as our's originally? Or shall we spread our knowledge broad-cast all over the land, and receive in turn all the knowledge, and improvements, and inventions, of the host of dentists by whom we are surrounded.

It requires but little reflection on the part of any one whose mind can be brought fairly to comprehend the results of the two systems, to decide which would be best for community; and very few, I think, will fail to perceive also, that the latter plan if fairly carried out, would conduce to the interest of each individual dentist.

All will admit this in the abstract, but *how* to have this universal distribution fairly carried out,—that's the question? Amid the uncertainty of results shall we dare to try experiments. Let those who hesitate, look back the last forty years, and see if the results of the old system have been such as to make them very tenacious of adhering to it, even if the new one should not remove all the evils nor confer all the benefits which theorists might anticipate.

Under the old system, when all kept their own secrets, and many supposed they were practicing that which no one else knew how to do, and thought that knowledge would die with themselves,—how common it was when any one in the generosity of his heart, condescended to communicate some improved *modus operandi*, to hear the response, "why, I have been doing that these twenty years!" How common too, the practice of dishonorable men, in claiming credit for improvements to which they well knew they had no just title; and yet, as these improvements had been kept as secrets by the real inventors, it was not easy always to disabuse the public, or render credit where it justly belonged. There seems to be a change going on in these respects. Dentists are placing themselves on record. *Associated effort*, even with all the disadvantages under which it still labors, has evidently begun to work a change for the better. The question is not so much now, when did you do this, or that, first? but, when did you first communicate it? not so much now, who did it first, as, who gave it first to the profession?

Slow as many of us have been to admit the benefits of dental associations (myself among the number), we can not longer shut our eyes to the fact, that progress has been made. The evils have been less, the benefits have been greater than were apprehended. The objects and the aims of these societies have sensibly improved. We all, or many of us at least, recollect the ridiculous positions assumed by some of the first organizations of this kind. The attempts to establish by edict what was, and what was not sound practice, and to affix pains and penalties thereto. The numerous raids preached up, from time to time, against quacks and quackery, most of which resulted in showing up the raiders to be the greatest quacks in question.

The amalgam question was one of these. It was as much as a man's professional life was worth, at one time, to admit that he used the article in his practice. I have known more than one good man, and very tolerable operator, driven away from a respectable practice, by some zealous opponent who could out-talk and out-write him, on the merits and demerits of this apple of discord, who, in after days, were said as far to out-herod Herod, as to buy the "nasty stuff" by the pound.

So far as these societies were attempted to be used for the purpose of abridging the liberty, controlling the judgment, or establishing compulsory tariffs of prices, they were all wrong; and resulted in evil, and only evil; and it was this tendency in the first organizations, to be converted into combinations for selfish purposes, that induced so many of the most discreet members of the profession to stand aloof. Perhaps it is well they did so, for the elements of discord were sooner burned out, and by common consent now, dentists meet in conventions, societies, or in smaller circles, and discuss the merits and demerits of the various modes of practice, without ever deeming it necessary to decide by vote which plan is best, or what practices are unprofessional, or in any other way attempting to curtail the liberty of any; relying solely on the effect of free communication and the interchange of opinions, and the results of the various modes of practice; leaving the mind of each individual to draw his own conclusions, and be guided by his own enlightened judgment. Societies thus conducted, enable every one, and make it his duty even, to communicate any new modes of practice, or improved manipulations to his associates.

The novelties may not be appreciated at the time, but the seed is sown, he has placed himself on record, and by and by, when one another have tried and approved the new thing, he is in little danger of seeing his lightning hitched to the car of another man's popularity, for the purpose of casting himself into the shade; or if the attempt is made, the thunderbolt recoils on the aggressor. I think we have reached a point where we can see light ahead. It seems to me that harmony is to arise out of discord.

That the whole profession will soon see that each individual member will be vastly the gainer by contributing his *mite*, and receiving *much* from the many in return. That the community will be better served by having the combined wisdom of all the dentists of the world, at the disposal of each, than it was when each individually was plodding along in the round of his own track, doing perhaps the best he could from the little store of experience he had laid up for himself from his own limited opportunities. But that this or any other plan will place all dentists on a level,—will pull down one or raise another up to his standard of usefulness, it would be utopian to suppose. It will elevate the eminent as much as it will raise the tyro in the profession.

While one will appropriate to his own use the wisdom of the wise, the other will profit by hearing rehearsed the failures and the trials of his less favored brethren. And the public then, as now, will reward every man according to the measure of his desert. Our duty will be discharged only, when we individually do the best we can, not only for our patients, but by opening wide the gates of access to any little stock of knowledge we may happen to possess, to all who aspire to join our ranks, and to fight under our banner, against the great host of diseases to which humanity is subject.

Allow me, gentlemen, to close these remarks by again expressing my thanks for the gentlemanly deportment which has always rendered our meetings so pleasant and agreeable; and also to assure you of my sincere good wishes for each of you individually—for your continued success and increased prosperity, as well as for your combined usefulness as a society.

